# Studying sex differences in responses to fibroblast growth factor 21 administration in obese mice consuming a sweet-fat diet

**DOI:** 10.18699/VJGB-23-40

**Published:** 2023-07

**Authors:** N.М. Bazhan, T.V. Jakovleva, A.Yu. Kazantseva, N.E. Kostina, P.E. Orlov, N.Yu. Balybina, K.О. Baranov, E.N. Makarova

**Affiliations:** Institute of Cytology and Genetics of the Siberian Branch of the Russian Academy of Sciences, Novosibirsk, Russia Novosibirsk State University, Novosibirsk, Russia; Institute of Cytology and Genetics of the Siberian Branch of the Russian Academy of Sciences, Novosibirsk, Russia; Institute of Cytology and Genetics of the Siberian Branch of the Russian Academy of Sciences, Novosibirsk, Russia; Institute of Cytology and Genetics of the Siberian Branch of the Russian Academy of Sciences, Novosibirsk, Russia; Novosibirsk State University, Novosibirsk, Russia; Institute of Cytology and Genetics of the Siberian Branch of the Russian Academy of Sciences, Novosibirsk, Russia; Institute of Molecular and Cellular Biology of the Siberian Branch of the Russian Academy of Sciences, Novosibirsk, Russia; Institute of Cytology and Genetics of the Siberian Branch of the Russian Academy of Sciences, Novosibirsk, Russia

**Keywords:** FGF21, obesity, sex differences, liver, adipose tissue, hypothalamus, food intake, gene expression, FGF21, ожирение, половые различия, печень, жировая ткань, гипоталамус, потребление пищи, экспрессия генов

## Abstract

In animals, obesity caused by consumption of a sweet-fat diet (SFD) is the most adequate mouse model of human diet-induced obesity. Fibroblast growth factor 21 (FGF21) reduces body weight, beneficially affects taste preferences, and corrects glucose metabolism in obese mice. Sex is known to influence FGF21 effects in different models of diet-induced and hereditary obesity. In mice with SFD-induced obesity, the effects of FGF21 have been studied only in males. The aim of this study was to compare the effects of FGF21 on body weight, food preferences and glucose and lipid metabolism in C57Bl/6J male and female mice with SFD-induced obesity. Mice were fed with a diet consisting of standard chow, lard and cookies for 10 weeks, then they were injected with FGF21 (1 mg per 1 kg) or vehicle for 7 days. Body weight, weights of different types of food, blood parameters, glucose tolerance, gene and protein expression in the liver, gene expression in the white, brown adipose tissues, and the hypothalamus were assessed. FGF21 administration reduced body weight, did not alter total energy consumption, and activated orexigenic pathways of hypothalamus in mice of both sexes. However, sex dimorphism was found in the realization of the orexigenic FGF21 action at the transcriptional level in the hypothalamus. Metabolic effects of FGF21 were also sex-specific. Only in males, FGF21 exerted beneficial antidiabetic action: it reduced fatty acid and leptin plasma levels, improved glucose-tolerance, and upregulated hepatic expression of Ppargc1, Fasn, Accα, involved in lipid turnover, gene Insr and protein glucokinase, involved in insulin action. Only in obese females, FGF21 induced preference of standard diet to sweet food. Thus, in mouse model of obesity induced by consumption of a sweet-fat diet, the catabolic effect of FGF21 was not sex-specific and hormonal, transcriptional and behavioral effects of FGF21 were sex-specific. These data suggest elaboration of different approaches to use FGF21 analogs for correction of metabolic consequences of obesity in different sexes.

## Abstract (ru)

У животных ожирение, вызванное потреблением сладко-жирной диеты, служит наиболее адекватной моделью развития алиментарного ожирения у человека. У мышей с ожирением фактор роста фибробластов 21 (FGF21) снижает массу тела, благоприятно влияет на вкусовые предпочтения, улучшает метаболизм глюкозы и липидов. В различных моделях ожирения показано, что пол влияет на эффекты FGF21. У мышей с ожирением, вызванным сладко-жирной диетой, эффекты FGF21 изучены только у самцов. Цель работы заключалась в сравнении влияния FGF21 на массу тела, выбор пищи, углеводно-жировой обмен у самцов и самок мышей C57Bl/6J с ожирением, вызванным потреблением сладко-жирной диеты. В течение 10 недель мышей кормили диетой, состоящей из стандартного лабораторного корма, свиного сала и сладкого печенья, затем 7 дней они получали инъекции FGF21 (1 мг на 1 кг) или растворителя. Измерены масса тела, масса потребленных продуктов, показатели крови, толерантность к глюкозе, экспрессия в печени генов и белков, а в жировой ткани и гипоталамусе – только генов. У мышей обоих полов введение FGF21 снизило массу тела, не изменило общее количество потребленной энергии и активировало орексигенные пути в гипоталамусе. Однако на транскрипционном уровне выявлены половые различия в реализации орексигенного действия FGF21 в гипоталамусе. Метаболическое действие FGF21 также зависело от пола. Только у самцов FGF21 вызывал благоприятное антидиабетическое воздействие: снижал уровни жирных кислот и лептина в крови, улучшал толерантность к глюкозе, повышал экспрессию в печени генов Ppargc1, Fasn, Accα, вовлеченных в жировой обмен, и гена Insr, а также белка глюкокиназы, которые опосредуют действие инсулина. Только у самок FGF21 увеличивал потребление энергии со стандартным кормом и снижал – с печеньем. Таким образом, у мышей в модели ожирения, вызванного сладко-жирной диетой, FGF21 уменьшал массу тела особей обоего пола, оказывал антидиабетический эффект только у самцов и менял вкусовые предпочтения только у самок. Эти результаты указывают на необходимость разработки специальных подходов в использовании FGF21 и его аналогов для лечения метаболических последствий ожирения у представителей разных полов.

## Introduction

In the human population, there is a significant increase in the number of people suffering from obesity and associated metabolic diseases such as type 2 diabetes, cardiovascular diseases and non-alcoholic fatty liver. Intensive research is underway to create drugs for normalization of carbohydrate-lipid metabolism at obesity. Fibroblast growth factor 21 (FGF21) is purported to be one of the most promising candidates for such aims (Talukdar, Kharitonenkov, 2021). FGF21 is synthesized and secreted into the circulation mainly by the liver (Fisher et al., 2011) in response to metabolic stresses such as food deprivation (Zhang Y. et al., 2012; Bazhan et al., 2019a), cold exposure (Dutchak et al., 2012), and obesity (Chukijrungroat et al., 2017; Bazhan et al., 2019b). FGF21 functions to restore homeostasis by coordinating metabolic responses from brown and white adipose tissues, muscles, liver and hypothalamus (Martínez-Garza et al., 2019; Makarova et al., 2021a, b). The effect of FGF21 on metabolic phenotype is partially mediated by its effect on gene expression in these tissues (Hale et al., 2012; Keinicke et al., 2020). FGF21 administration has potent beneficial effects on obesity and diabetes in humans, monkeys, and rodents (Kharitonenkov, Adams, 2013), it reduces body weight, increases insulin sensitivity, normalizes blood glucose levels (Kharitonenkov et al., 2005; Coskun et al., 2008; Xu et al., 2009).

High energy density diets: high fat diet (HFD) and sweet fat diet (SFD) are most commonly used to induce obesity in mice. It has recently been shown that the anti-diabetic effect of FGF21 in genetically obese mice (ob/ob and Ay mice) appears only in males (Berglund et al., 2009; Makarova et al., 2020; Makarova et al., 2021b), whereas in mice with obesity caused by HFD it is manifested both in females and males. In mice with SFD-induced obesity (SFDIO), the effects of FGF21 have been shown in males (Coskun et al., 2008), while in females, they have not been studied. Elucidation of the role of FGF21 in the regulation of metabolic processes in females fed SFD is relevant, since SFD mimics the human Western diet and is the most adequate model of human diet-induced obesity (Sampey et al., 2011)

In addition, FGF21 may influence food choices. In mice with normal body weight, FGF21 administration has been shown to increase protein intake (Hill et al., 2020) and reduce sugar intake (Talukdar et al., 2016b). In ovariectomized obese female mice fed mixed diet (standard chows, lard and sweet cookies), FGF21 administration increases the intake of calories with standard chows, which contain more proteins than other types of food (Jakovleva et al., 2022). It is not known whether FGF21 influences the intake of various types of food and the expression of hypothalamic genes involved in the regulation of food intake in SFDIO mice with normal gonadal function.

Thus, the aim of this work was to study in male and female mice with obesity caused by the consumption of a sweet-fat diet the effect of FGF21 on weight of body, liver, adipose tissues, tolerance to glucose, blood levels of hormones and metabolites, the amount of energy consumed with various types of food, and expression of genes involved in the regulation of metabolic processes in the liver, white, brown adipose tissue and in the regulation of eating behavior in the hypothalamus.

## Materials and methods

The experiment was performed according to the European Convention for the Protection of Vertebrate Animals used for Experimental and other Scientific Purposes (Council of Europe No 123, Strasbourg 1985) and Russian national instructions for the care and use of laboratory animals. The protocols were approved by the Independent Ethics Committee of the Institute of Cytology and Genetics of the Siberian Branch of the Russian Academy of Sciences on November 8, 2021.

Animals. Male and female C57BL mice bread in the vivarium of the Institute of Cytology and Genetics were used. At the age of four weeks, mice were separated from mothers and placed in groups of three per cage under a 12/12-h light-dark regime (light from 07:30 to 19:30) at an ambient temperature of 22–24 °C. The animals were provided ad libitum ac cess to commercial mouse chow (Assortiment Agro, Turakovo Village, Moscow region, Russia) and water. At the age of 10 weeks, lard and sweet butter cookies were added to the standard chow in order to induce the development of obesity. The animals fed SFD for 13 weeks until they reached marked obesity (the body weight was 41.6 ± 1.0 g, mean ± SE, n = 20). To measure the food intake of each mouse, they were placed in separate cages and kept individually until the start of the experiment. Solitary maintenance is emotional stress, reducing body weight. Three days after the cage change, the body weight decreased to 38.9 ± 0.9 g. Two weeks after the cage change, the weight of the mice recovered to 41.8 ± 0.8 g. This body weight was considered as the initial.

Mice of both sexes were randomized into the control group injected with vehicle – phosphate-bicarbonate solution – and the FGF21 group injected with mouse recombinant FGF21 (1 mg per 1 kg). Each group consisted of 5–7 mice. Substances were administered subcutaneously at the end of the light period (17:00–17:30) for seven days. The protocol of the expression and purification of mouse FGF21 was described previously (Makarova et al., 2021a). Mice were weighed daily. In the part of the animals, various food components were also weighed daily and the amount of energy intake was calculated based on the fact that the calorie content of lard is 8 kcal/g, the standard chow is 2.5 kcal/g, and the biscuit is 4.58 kcal/g. The percentage of consumed energy in relation to total consumed energy was calculated for each type of food.

A day after the last injection of FGF21, some of the mice were sacrificed by decapitation, and the others were tested for glucose tolerance and were sacrificed the day after testing. Trunk blood was collected in test tubes with EDTA after decapitation, centrifuged and plasma was stored at –20 °C until the assay of hormones and metabolites. Liver, subcutaneous and abdominal white adipose tissue (WAT), and brown adipose tissue (BAT) from the interscapular region were weighed. Samples of the liver, BAT, perigonadal WAT and hypothalamus were collected and snap-frozen in liquid nitrogen to measure gene expression. In the liver, protein expression was also measured by Western Blot Analysis.

Glucose tolerance test. Before the test, food was removed from the animals at 08:00, and the test started at 15:00. Animals were injected with glucose (AO “REACHEM”, Moscow, Russia) intraperitoneally at the dose of 1 g/kg body weight. Blood glucose concentrations were measured using a Lifescan One Touch Basic Plus glucometer (LifeScan Inc., Switzerland) before glucose administration (fasting glucose) and 15, 30, 60, and 120 minutes after glucose administration

Assay of plasma biochemical parameters. Concentrations of insulin, leptin, and adiponectin were measured using a Rat/Mouse insulin ELISA Kit, a Mouse leptin ELISA Kit (EMD Millipore, St. Charles, MO, USA), and a Mouse adiponectin ELISA Kit (EMD Millipore, Billerica, MA, USA), respectively. Concentrations of glucose, triglycerides, and cholesterol were measured colorimetrically using Fluitest GLU, Fluitest TG, and Fluitest CHOL (Analyticon® Biotechnologies AGAm Mühlenberg 10, 35104 Lichtenfels, Germany), respectively. Concentrations of free fatty acids were measured using NEFA FS DiaSys kits (DiaSys Diagnostic Systems GmbH, Holzheim, Germany).

Relative quantitation real-time PCR. RNA was isolated from tissue samples using an ExtractRNA kit (Evrogen, Moscow, Russia) according to the manufacturer’s recommendations. First-strand cDNA was synthesized using Moloney murine leukemia virus (MMLV) reverse transcriptase (Evrogen) and oligo(dT) as a primer. TaqMan gene expression assays (Applied Biosystems, USA) were used for relative quantitation real-time PCR. The genes tested involved acetyl-CoA carboxylase alpha/beta (Acacα/β, Mm01304257_m1/ Mm01204671_ m1), adipose triglyceride lipase (Atgl, Mm00503040_m1), agouti related neuropeptide (Agrp, Mm00475829_g1), betaactin (Actb, Mm00607939_s1), carnitine palmitoyltransferase 1A/1B (Cpt1α/β, Mm01231183_m1/Mm00487191_g1), corticotrophin releasing hormone (Crh, Mm01293920_s1), fatty acid synthase (Fasn, Mm00662319_m1), fibroblast growth factor 21 (Fg f 21, Mm00840165_g1), glucose-6-phosphatase (G6pc, Mm00839363_m1), Glucokinase (Gck, Mm00439129_ m1), Insulin receptor (Insr, Mm01211875_m1), hormonesensitive lipase (Lipe, Mm00495359_m1), klotho beta (Klb, Mm00473122_m1), neuropeptide Y (Npy, Mm01410146_ m1), peroxisome proliferator-activated receptor alpha/gamma (Pparα/γ, Mm00440939_m1/Mm00440940_m1), peroxisome proliferator-activated receptor gamma coactivator (Ppargc1α, Mm01208835_m1), phosphoenolpyruvate carboxykinase (Pck, Mm01247058_m1), Pyruvate kinase liver and red blood cell (Pklr, Mm00443090_m1), proopiomelanocortin (Pomc, Mm00435874_m1), solute carrier family 2 member 1/2/4 (Slc2a1/Slc2a2 Slc2a4, Mm00441480_m1/Mm00446229_ m1/Mm00436615_m1), uncoupling protein 1/3 (Ucp1/Ucp3, Mm01244861_m1/ Mm01163394_m1). Beta-actin was used as endogenous controls. The PCR and fluorescence detection were performed on an Applied Biosystems VIIA 7 Real-Time PCR System. Relative quantification was performed by the comparative threshold cycle (CT) method (i. e. 2−ΔΔCt method).

Western Blot Analysis of protein levels. Expression of hepatic proteins was measured as described previously (Iakovleva et al., 2020). Primary polyclonal rabbit antibodies (Santa Cruz Biotechnology, USA, breeding 1:2000) were used: Insulin receptor antibody (IRa, N-20) and Glucokinase antibody (GK H-88). The results referred to the total amount of protein

Statistical analysis. Each result is presented as mean ± SE for a sample size (i. e., number of mice) indicated. A repeated measures ANOVA with the factors “sex” (male, female), “experiment” (phosphate-bicarbonate solution, FGF21), and “day of experiment” was used to analyze FGF21 effects on body weight loss and total daily energy intake. Other parameters were compared with Student’s t-test. Significance was determined as p < 0.05. The STATISTICA 6 software package (StatSoft Inc., USA) was used for analysis.

## Results

**Fig. 1.**
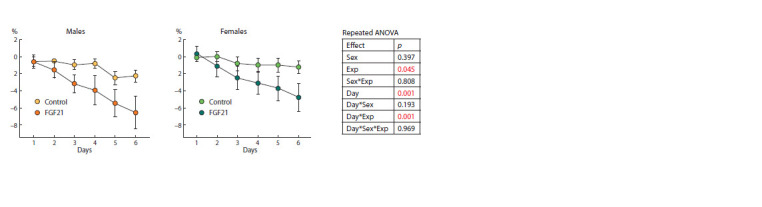
Body weight loss (% of initial BW) in mice with sweet fat diet-induced obesity received vehicle (control) or FGF21. Group size 5–7 animals.

Weight characteristics. Injections of vehicle and FGF21 induced weight loss in both male and female mice ( p <0.05, factor “experiment”; Fig. 1). However, weight loss was more pronounced in FGF21 than in the control group ( p < 0.001, interaction of “experiment” and “day of experiment” by repeated measures ANOVA) regardless of sex.

Sex and FGF21 administration did not affect indexes of the liver and adipose tissues (Table 1).

**Table 1.**
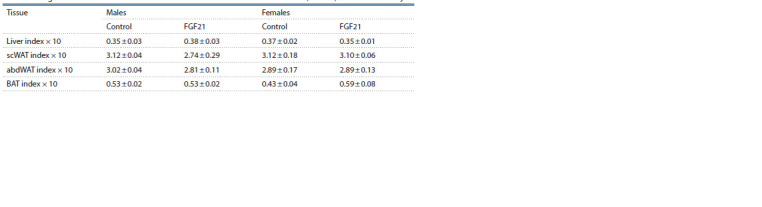
Weight characteristics in male and female obese mice that received vehicle (control) or FGF21 for 7 days Note. scWAT, subcutaneous white adipose tissue; abdWAT, abdominal white adipose tissue; BAT, brown adipose tissue.

**Fig. 2.**
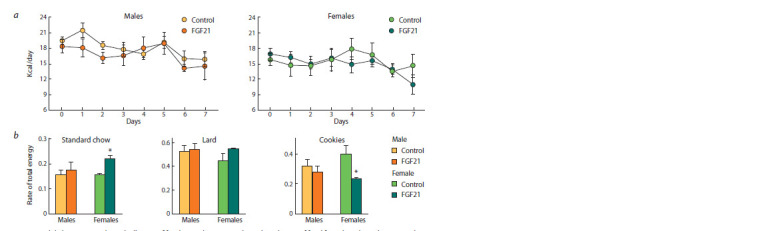
Total daily energy intake with all types of food (a) and energy intake with each type of food for 7 days (b) in obese mice that received vehicle (control) or FGF21 for 7 days. 5–7 mice/group. * p < 0.05 vs. control in the same sex by Student’s t-test.

Energy consumption. In both groups, males and females did not differ in total energy consumption with different types of food. FGF21 administration did not affect total energy intake or individual food choices in males, but increased energy intake with standard food and decreased it with cookies ( p < 0.05 in both cases) in females (Fig. 2, a, b). At the same time, in females treated with FGF21, the total energy intake from all types of food did not differ from that in the control group.

Blood biochemical parameters, glucose tolerance test. In the control group, no sex differences were found for most of the hormonal and metabolic blood parameters, only the adiponectin blood level in the females was higher than that of males ( p <0.001).

**Fig. 3.**
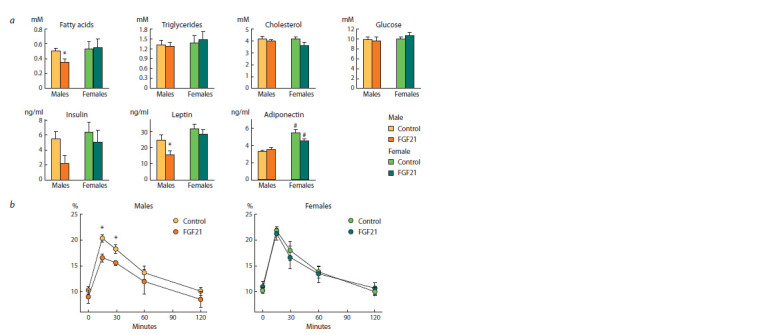
Biochemical blood parameters (a) and blood glucose levels in the glucose tolerance test (b) in obese mice that received vehicle (control) or FGF21. Here and in Fig. 4–7: 5–7 mice/group. * p < 0.05 vs. control in the same sex, # p < 0.05 vs. males in the same experimental group by Student’s t-test.

FGF21 administration reduced blood free fatty acid and leptin concentrations ( p < 0.05 in both cases), tended to reduce insulin concentration ( p <0.08) (Fig. 3, a) and increased glucose tolerance only in males (see Fig. 3, b). In the glucose tolerance test in males treated with FGF21, the blood glucose curve was lower than in the control, and at the 15th and 30th minute of the test, the differences with the control were significant (* p < 0.05 vs. control in both cases).

**Fig. 4.**
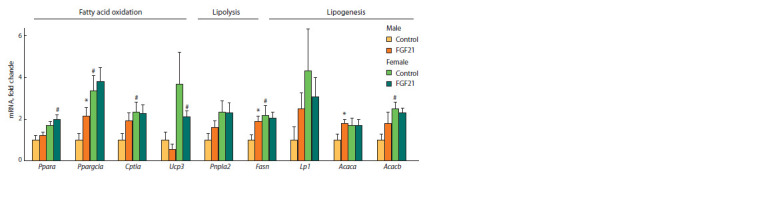
Relative expression of hepatic genes involved in lipid metabolism in obese mice that received vehicle (control) or FGF21.

**Fig. 5.**
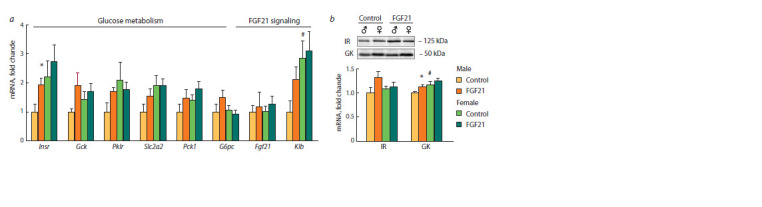
Relative expression of hepatic genes (a) and proteins (b) involved in glucose metabolism and FGF21 signaling in male and female obese mice that received vehicle (control) or FGF21.

Gene expression. In the control group, the expression of many hepatic genes in females was higher than in males (Fig. 4, 5). These include genes encoding peroxisome proliferator- activated receptor gamma coactivator (Ppargc1), carnitine palmitoyltransferase 1A (Cpt1α), fatty acid synthase (Fasn), acetyl-CoA carboxylase beta (Acacβ) and klotho beta (Klb) ( p <0.01 for Acacβ, and p < 0.05 for other genes). In addition, the expression of adipose triglyceride lipase (Pnpla2), solute carrier family 2 member 2 (Slc2a2), insulin receptor (Insr) genes in control females was higher than in SFDIO males at the trend level ( p = 0.06 for Pnpla2, and p = 0.07 for Insr and Slc2a2) (see Fig. 4).

Only in males, administration of FGF21 increased expression of hepatic genes involved in fatty acid oxidation (Ppargc1), lipogenesis (Fasn, acetyl-CoA carboxylase α, Acacα) and insulin sensitivity (Insr) ( p < 0.05 for all genes) (see Fig. 4, 5). In addition, in SFDIO males, FGF21 increased, on a tendency level, the expression of genes related to glucose oxidation (glucokinase, Gck, pyruvate kinase, Pklr) ( p < 0.06 for Pklr, and p < 0.07 for Gck). After FGF21 administration, the expression of genes involved in fatty acid oxidation (peroxisome proliferator-activated receptor alpha, Pparα and uncoupling protein 3, Ucp3) was higher in females than in males ( p < 0.05 for both genes).

As far as FGF21 administration upregulated the expression of genes encoding insulin receptor (IR) and glucokinase (GK), hepatic expression of these proteins was also measured (see Fig. 5, b). In the control group, IR expression did not differ between females and males and GK expression was higher in females than in males ( p <0.05). FGF21 administration increased expression of GK ( p < 0.05) and IR (tendency p < 0.06) only in males.

**Fig. 6.**
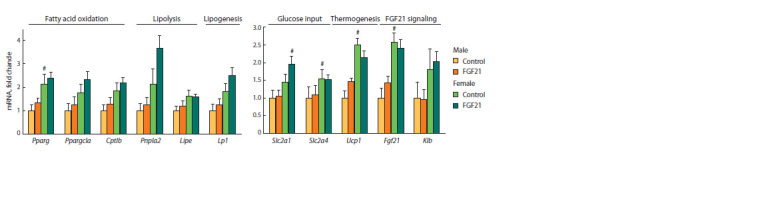
Relative BAT gene expression in male and female obese mice that received vehicle (control) or FGF21

**Fig. 7.**
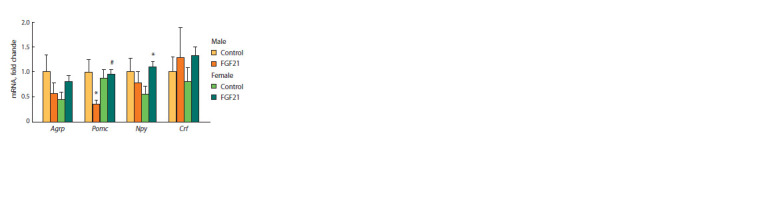
Relative hypothalamic gene expression in male and female obese mice that received vehicle (control) or FGF21.

In obese mice from the control group, expression of BAT genes involved in fatty acid oxidation (peroxisome proliferator- activated receptor gamma, Pparγ), thermogenesis (uncoupling protein 1, Ucp1) and gene encoding FGF21 (Fgf21) was higher in females than in males ( p < 0.001 for Fgf21, p <0.01 for Ucp1, and p <0.05 for Pparγ) (Fig. 6). FGF21 administration did not affect BAT gene expression. After FGF21 administration expression of Slc2a1 encoding Solute Fig. 7. Relative hypothalamic gene expression in male and female obese mice that received vehicle (control) or FGF21. carrier family 2 member 1 was significantly higher in females than in males ( p < 0.05). In subcutaneous WAT, expression of all measured genes (Pparγ, Ppargc1, Cpt1β, Pnpla2, Lipe, Lpl, Fasn, Slc2a1, Slc2a4, Ucp1, Insr) was independent of sex and FGF21 administration (the data are not presented).

In the control group, males and females did not differ in the expression of hypothalamic genes involved in the regulation of food intake (Fig. 7). FGF21 administration increased the expression of the gene encoding orexigenic neuropeptide NPY in SFDIO females and decreased the expression of gene encoding anorexigenic neuropeptide POMC in SFDIO males ( p < 0.05 for both genes). In the FGF21 group, the expression of the Pomc gene in females was higher than in males ( p < 0.05).

## Discussion

In this work, we evaluated the pharmacological effects of FGF21 in male and female mice in a model of obesity induced by consumption of SFD that mimics the human Western diet and is the most adequate model of human diet-induced obesity (Sampey et al., 2011). Results of this work demonstrated that in C57Bl mice with SFDIO, FGF21 exerted catabolic effect regardless of sex, and antidiabetic effect – differently in male and female mice. FGF21 administration exerted anti-diabetic therapeutic effects only in SFDIO males and increased consumption of standard chow and decreased consumption of sweet cakes only in SFDIO females.

The ability of FGF21 to reduce body weight, regardless of sex, was previously demonstrated in obese mice that consumed HFD (Makarova et al., 2021a). However, in Ay mice with genetic melanocortin obesity, the catabolic effect of FGF21 was manifested only in males (Makarova et al., 2020). Thus, FGF21-treated obese male mice exert reduced BW regardless of the type of obesity (hereditary, HFD- or SFD-induced obesity), and FGF21-treated obese female mice – only at alimentary form of obesity.

In SFDIO mice, the catabolic effect of FGF21 was not associated with a decrease in total energy intake, suggesting that it was due to an increase in energy expenditure. Indeed, T. Coskun et al. demonstrated that FGF21-treated male mice with obesity induced by consumption of a sweet-fat diet had a significantly higher energy expenditure rate, oxygen consumption as well as increased core body temperature, compared with the vehicle-treated mice (Coskun et al., 2008). The authors hypothesized that the FGF21-induced increase in energy expenditure could be due to increased thermogenesis in BAT, since the expression of genes involved in thermogenesis and fatty acid oxidation was increased in FGF21-treated male mice. In our experiment, FGF21 administration did not alter expression of genes (Ucp1 and Dio2) related to thermogenesis in BAT and WAT in SFDIO mice. It can be assumed that in SFDIO mice, FGF21 increased energy expenditure through other mechanisms, unrelated to BAT and WAT thermogenesis.

It is known that FGF21 enhances mitochondrial biogenesis and fatty acid oxidation not only in adipose tissues, but also in muscles (Sun et al., 2021). In addition, FGF21 can increase energy expenditure by activating sympathetic nerves (Owen et al., 2014), which is accompanied by an increase in body temperature and physical activity (Owen et al., 2014). We demonstrated earlier that FGF21 increases locomotor activity equally in male and female Ay mice with genetic obesity (Makarova et al., 2021a). It can be assumed that the catabolic effect of FGF21 in SFDIO mice was due to activation of thermogenesis in muscles (Sun et al., 2021), and motor activity (Makarova et al., 2021a). Weight loss in FGF21-treated SFDIO mice is indicative of yet other mechanisms whereby energy expenditure may be increased, which remain to be explored.

The antiobesity effect of FGF21 was associated with activation of hypothalamic orexigenic mechanisms in mice of both sexes. However, sex dimorphism was found in the realization of the orexigenic FGF21 action at the transcriptional level: FGF21 upregulated the expression of the orexigenic Npy in SFDIO females, and downregulated the expression of the anorexigenic Pomc in SFDIO males. These data suggest that specific pathways for the orexigenic action of FGF21 may differ between males and females. Obviously, transcriptional changes found at the level of the hypothalamus do not contribute to body weight loss in response to FGF21 administra-tion. It can be assumed that they are part of a counter-regulatory mechanism necessary for limiting the catabolic effect of FGF21.

Our data demonstrated that FGF21 had a beneficial antidiabetic effect only in SFDIO males: it decreased plasma FFA, leptin levels, tended to reduce plasma insulin level, and increased glucose tolerance. Sex dimorphism in response to FGF21 was reported earlier in obese Ay mice: FGF21 administration decreases hyperinsulinemia and hepatic lipid accumulation, increases muscle expression of genes involved in fatty acid oxidation and insulin signaling only in obese Ay males (Makarova et al., 2021b). It is possible that in SFDIO males, the FGF21-induced improvement in glucose tolerance was associated with a decrease in plasma concentration of leptin and fatty acids which are key risk factors for insulin resistance at obesity (Yang et al., 2018; Zhang Q. et al., 2019). Zhao and coauthors, using genetic approaches and a leptin neutralizing antibody, demonstrated that in obese mice, a partial reduction of plasma leptin levels restores hypothalamic leptin sensitivity and effectively enhances glucose tolerance and insulin sensitivity (Zhao et al., 2019).

In SFDIO males, the antidiabetic effect of FGF21 is most likely due to its action on the liver, which is a highly dimorphic target organ for FGF21 and plays a key role in the regulation of carbohydrate and lipid metabolism (Fisher et al., 2011; Torre et al., 2017). In our study, FGF21-treated SFDIO males exerted upregulated expression of hepatic genes (Pclr, Gck – both tendency), and proteins (GK and IR – tendency) involved in insulin signaling and genes related to fatty acid oxidation (Ppargc1) and lipogenesis (Fasn, Acacα), suggesting increased glucose and lipid turnover

In SFDIO females, FGF21 administration did not increase the expression of any hepatic gene, which could be due to a ceiling effect. In vehicle-treated SFDIO mice, expression of many hepatic genes involved in lipid metabolism (Ppargc1a, Cpt1, Fasn, Accb) and expression of GK was higher in females than in males. It can be assumed that the increased expression of hepatic genes in vehicle-treated SFDIO females is at least partially due to the influence of estradiol, the expression of its receptors in the liver of female mice is significantly higher than in male mice (Torre et al., 2017). Estradiol administration to ovariectomized obese female mice increases expression of the insulin receptor gene (Insr) and uncoupling protein 2 gene (Ucp2) in the liver (Jakovleva et al., 2022). The relatively high initial level of gene expression could not be further enhanced by FGF21 administration (ceiling effect).

In addition, FGF21 and estradiol can interact with each other at the level of intracellular signal transduction pathways. According to the available data, estradiol and FGF21 have different receptors and the same signaling pathways (Fisher et al., 2011; Vrtačnik et al., 2014). Recently we demonstrated that in ovariectomized obese female mice, FGF21 reduced plasma insulin level, expression of Pklr and upregulated expression of Irs2 in the liver. FGF21 did not affect these parameters if it was administered together with estradiol (Jakovleva et al., 2022). Thus, lack of the effect of FGF21 on hepatic gene expression in SFDIO females may be associated with crosstalk between estradiol and FGF21 in their actions

In normal-weight male mice, FGF21 is known to increase protein intake, decrease sugar and alcohol intake, and have no effect on total energy intake (Talukdar et al., 2016a; Hill et al., 2020). Until now, it was unknown to what extent the effect of FGF21 on taste preferences is reproduced in mice with SFD-induced obesity. We demonstrated that FGF21 did not affect the total calorie intake in SFDIO males and females, and affected taste preferences only in SFDIO females: it increased the intake of calories with standard food, decreased it with cookies and did not alter it with lard. The standard chow contained the maximum amount of proteins, compared with lard and cookies, and cookies contained a large amount of both carbohydrates and fats. Most likely, FGF21 reduced carbohydrate intake rather than lipid intake in SFDIO females, as it reduces sugar intake, but does not affect fat intake in experiments with a choice between two diets (Hill et al., 2020). Summing up, it can be assumed that FGF21 increased protein intake and reduced sweets intake in SFDIO female mice

The selective influence of FGF21 on the feeding behavior of SFDIO females could be due to estrogen action. Recently we demonstrated that in ovariectomized SFDIO female mice, FGF21 upregulates the expression of hypothalamic genes encoding leptin receptor (Lepr) and its own co-receptor β-klotho (Klb) and estradiol increases its stimulating effect (Jakovleva et al., 2022). These data suggest that estradiol may enhance the stimulatory effect of FGF21 on hypothalamic sensitivity to leptin and FGF21. It was reported that hypothalamic circuitry connects with CNS circuitry modulating feeding behavior and leptin inhibits motivated behavior for palatable food (Figlewicz et al., 2003).

Summing up the data of the present work and our previous studies, one can say that different high-energy obesogenic diets create different metabolic backgrounds, which may affect the effectiveness of FGF21 treatment in obese female mice: the antidiabetic FGF21 action was manifested if they were fed a high fat diet (Makarova et al., 2021b) and was not manifested if they were fed a diet containing a sweet component along with fat. The mechanisms of diet-dependent action of FGF21 in females require special study, since they may affect the tactics of using FGF21 in individuals of different sexes.

## Conclusion

Our study demonstrated that in SFDIO mice, FGF21 reduced body weight, did not alter total energy consumption, and activated orexigenic pathways of hypothalamus in mice of both sexes. However, sex dimorphism was found in the realization of the orexigenic FGF21 action at the transcriptional level in the hypothalamus. Hormonal, transcriptional and behavioral responses to FGF21 were also sex-specific. Only in SFDIO males, therapeutic effects of FGF21 administration – a decrease in fatty acid and leptin plasma levels, improvement of glucose tolerance, and upregulation of expression of genes involved in insulin signaling and lipid turnover in the liver – were observed. Only in SFDIO females, FGF21 induced preference of standard diet to sweet food.

## Conflict of interest

The authors declare no conflict of interest.

## References

Bazhan N., Jakovleva T., Feofanova N., Denisova E., Dubinina A.,
Sitnikova N., Makarova E. Sex differences in liver, adipose tissue,
and muscle transcriptional response to fasting and refeeding in mice.
Cells. 2019a;8(12):1529. DOI 10.3390/cells8121529.

Bazhan N., Jakovleva T., Balyibina N., Dubinina A., Denisova E.,
Feofanova N., Makarova E. Sex dimorphism in the Fgf21 gene expression
in liver and adipose tissues is dependent on the metabolic
condition. OnLine J. Biol. Sci. 2019b;19(1):28-36. DOI 10.3844/
ojbsci.2019.28.36.

Berglund E.D., Li C.Y., Bina H.A., Lynes S.E., Michael M.D., Shanafelt
A.B., Kharitonenkov A., Wasserman D.H. Fibroblast growth
factor
21 controls glycemia via regulation of hepatic glucose flux
and insulin sensitivity. Endocrinology. 2009;150(9):4084-4093. DOI
10.1210/en.2009-0221

Chukijrungroat N., Khamphaya T., Weerachayaphorn J., Songserm T.,
Saengsirisuwan V. Hepatic FGF21 mediates sex differences in highfat
high-fructose diet-induced fatty liver. Am. J. Physiol. Endocrinol.
Metab. 2017;313(2):E203-E212. DOI 10.1152/ajpendo.00076.
2017.

Coskun T., Bina H.A., Schneider M.A., Dunbar J.D., Hu C.C., Chen Y.,
Moller D.E., Kharitonenkov A. Fibroblast growth factor 21 corrects
obesity in mice. Endocrinology. 2008;149(12):6018-6027. DOI
10.1210/en.2008-0816

Dutchak P.A., Katafuchi T., Bookout A.L., Choi J.H., Yu R.T., Mangelsdorf
D.J., Kliewer S.A. Fibroblast growth factor-21 regulates
PPARγ activity and the antidiabetic actions of thiazolidinediones.
Cell. 2012;148(3):556-567. DOI 10.1016/j.cell.2011.11.062

Figlewicz D.P., Evans S.B., Murphy J., Hoen M., Baskin D.G. Expression
of receptors for insulin and leptin in the ventral tegmental area/
substantia nigra (VTA/SN) of the rat. Brain Res. 2003;964(1):107-
115. DOI 10.1016/s0006-8993(02)04087-8.

Fisher F.M., Estall J.L., Adams A.C., Antonellis P.J., Bina H.A.,
Flier
J.S., Kharitonenkov A., Spiegelman B.M., Maratos-Flier E.
Integrated regulation of hepatic metabolism by fibroblast growth
factor 21 (FGF21) in vivo. Endocrinology. 2011;152(8):2996-3004.
DOI 10.1210/en.2011-0281.

Hale C., Chen M.M., Stanislaus S., Chinookoswong N., Hager T.,
Wang M., Véniant M.M., Xu J. Lack of overt FGF21 resistance in
two mouse models of obesity and insulin resistance. Endocrinology.
2012;153(1):69-80. DOI 10.1210/en.2010-1262.

Hill C.M., Qualls-Creekmore E., Berthoud H.R., Soto P., Yu S., McDougal
D.H., Münzberg H., Morrison C.D. FGF21 and the physiological
regulation of macronutrient preference. Endocrinology. 2020;
161(3):bqaa019. DOI 10.1210/endocr/bqaa019.

Iakovleva T.V., Kostina N.E., Makarova E.N., Bazhan N.M. Effect of
gonadectomy and estradiol on the expression of insulin signaling
cascade genes in female and male mice. Vavilovskii Zhurnal Genetiki
i Selektsii = Vavilov Journal of Genetics and Breeding. 2020;
24(4):427-434. DOI 10.18699/VJ20.635

Jakovleva T.V., Kazantseva A.Y., Dubinina A.D., Balybina N.Y., Baranov
K.O., Makarova E.N., Bazhan N.M. Estradiol-dependent and
independent effects of FGF21 in obese female mice. Vavilovskii
Zhurnal Genetiki i Selektsii = Vavilov Journal of Genetics and
Breeding. 2022;26(2):159-168. DOI 10.18699/VJGB-22-20.

Keinicke H., Sun G., Mentzel C.M.J., Fredholm M., John L.M., Andersen
B., Raun K., Kjaergaard M. FGF21 regulates hepatic metabolic
pathways to improve steatosis and inflammation. Endocr. Connect.
2020;9(8):755-768. DOI 10.1530/EC-20-0152.

Kharitonenkov A., Shiyanova T.L., Koester A., Ford A.M., Micanovic
R., Galbreath E.J., Sandusky G.E., Hammond L.J., Moyers J.S.,
Owens R.A., Gromada J., Brozinick J.T., Hawkins E.D., Wroblewski
V.J., Li D.S., Mehrbod F., Jaskunas S.R., Shanafelt A.B. FGF-21
as a novel metabolic regulator. J. Clin. Invest. 2005;115(6):1627-
1635. DOI 10.1172/JCI23606

Kharitonenkov A., Adams A.C. Inventing new medicines: The FGF21
story. Mol. Metab. 2013;3(3):221-229. DOI 10.1016/j.molmet.2013.
12.003

Makarova E., Kazantseva A., Dubinina A., Denisova E., Jakovleva T.,
Balybina N., Bgatova N., Baranov K., Bazhan N. Fibroblast growth
factor 21 (FGF21) administration sex-specifically affects blood insulin
levels and liver steatosis in obese Ay mice. Cells. 2021a;10(12):
3440. DOI 10.3390/cells10123440.

Makarova E., Kazantseva A., Dubinina A., Jakovleva T., Balybina N.,
Baranov K., Bazhan N. The same metabolic response to FGF21 administration
in male and female obese mice is accompanied by sexspecific
changes in adipose tissue gene expression. Int. J. Mol. Sci.
2021b;22(19):10561. DOI 10.3390/ijms221910561

Makarova E.N., Yakovleva T.V., Balyibina N.Y., Baranov K.O., Denisova
E.I., Dubinina A.D., Feofanova N.A., Bazhan N.M. Pharmacological
effects of fibroblast growth factor 21 are sex-specific in
mice with the lethal yellow (Ay) mutation. Vavilovskii Zhurnal Genetiki
i Selektsii = Vavilov Journal of Genetics and Breeding. 2020;
24(2):200-208. DOI 10.18699/VJ20.40-o.

Martínez-Garza Ú., Torres-Oteros D., Yarritu-Gallego A., Marrero P.F.,
Haro D., Relat J. Fibroblast growth factor 21 and the adaptive response
to nutritional challenges. Int. J. Mol. Sci. 2019;20(19):4692.
DOI 10.3390/ijms20194692

Owen B.M., Ding X., Morgan D.A., Coate K.C., Bookout A.L., Rahmouni
K., Kliewer S.A., Mangelsdorf D.J. FGF21 acts centrally to
induce sympathetic nerve activity, energy expenditure, and weight
loss. Cell Metab. 2014;20(4):670-677. DOI 10.1016/j.cmet.2014.
07.012.

Sampey B.P., Vanhoose A.M., Winfield H.M., Freemerman A.J.,
Muehlbauer
M.J., Fueger P.T., Newgard C.B., Makowski L. Cafeteria
diet is a robust model of human metabolic syndrome with
liver and adipose inflammation: comparison to high-fat diet. Obesity
(Silver Spring). 2011;19(6):1109-1117. DOI 10.1038/oby.2011.18.

Sun H., Sherrier M., Li H. Skeletal muscle and bone – emerging targets
of fibroblast growth factor-21. Front. Physiol. 2021;12:625287.
DOI 10.3389/fphys.2021.625287.

Talukdar S., Kharitonenkov A. FGF19 and FGF21: In NASH we
trust. Mol. Metab. 2021;46:101152. DOI 10.1016/j.molmet.2020.
101152

Talukdar S., Owen B.M., Song P., Hernandez G., Zhang Y., Zhou Y.,
Scott W.T., Paratala B., Turner T., Smith A., Bernardo B., Müller C.P.,
Tang H., Mangelsdorf D.J., Goodwin B., Kliewer S.A. FGF21 regulates
sweet and alcohol preference. Cell Metab. 2016a;23(2):344-
349. DOI 10.1016/j.cmet.2015.12.008.

Talukdar S., Zhou Y., Li D., Rossulek M., Dong J., Somayaji V.,
Weng Y., Clark R., Lanba A., Owen B.M., Brenner M.B., Trimmer
J.K., Gropp K.E., Chabot J.R., Erion D.M., Rolph T.P., Goodwin
B., Calle R.A. A long-acting FGF21 molecule, PF-05231023,
decreases body weight and improves lipid profile in non-human primates
and type 2 diabetic subjects. Cell Metab. 2016b;23(3):427-
440. DOI 10.1016/j.cmet.2016.02.001

Torre D., Lolli F., Ciana P., Maggi A. Sexual Dimorphism and Estrogen
Action in Mouse Liver [published correction appears in Adv. Exp.
Med. Biol. 2017;1043:E1]. Adv. Exp. Med. Biol. 2017;1043:141-
151. DOI 10.1007/978-3-319-70178-3_8.

Vrtačnik P., Ostanek B., Mencej-Bedrač S., Marc J. The many faces of
estrogen signaling. Biochem. Med. (Zagreb). 2014;24(3):329-342.
DOI 10.11613/BM.2014.035.

Xu J., Stanislaus S., Chinookoswong N., Lau Y.Y., Hager T., Patel J.,
Ge H., Weiszmann J., Lu S.C., Graham M., Busby J., Hecht R.,
Li Y.S., Li Y., Lindberg R., Véniant M.M. Acute glucose-lowering
and insulin-sensitizing action of FGF21 in insulin-resistant mouse
models–association with liver and adipose tissue effects. Am. J. Physiol.
Endocrinol. Metab. 2009;297(5):E1105-E1114. DOI 10.1152/
ajpendo.00348.2009

Yang Q., Vijayakumar A., Kahn B.B. Metabolites as regulators of insulin
sensitivity and metabolism. Nat. Rev. Mol. Cell Biol. 2018;
19(10):654-672. DOI 10.1038/s41580-018-0044-8.

Zhang Q., Kong X., Yuan H., Guan H., Li Y., Niu Y. Mangiferin improved
Palmitate-induced-insulin resistance by promoting free fatty
acid metabolism in HepG2 and C2C12 cells via PPARα: mangiferin
improved insulin resistance. J. Diabetes Res. 2019;2019:2052675.
DOI 10.1155/2019/2052675

Zhang Y., Xie Y., Berglund E.D., Coate K.C., He T.T., Katafuchi T.,
Xiao G., Potthoff M.J., Wei W., Wan Y., Yu R.T., Evans R.M.,
Kliewer S.A., Mangelsdorf D.J. The starvation hormone, fibroblast
growth factor-21, extends lifespan in mice. Elife. 2012;1:e00065.
DOI 10.7554/eLife.00065

Zhao S., Zhu Y., Schultz R.D., Li N., He Z., Zhang Z., Caron A., Zhu Q.,
Sun K., Xiong W., Deng H., Sun J., Deng Y., Kim M., Lee C.E., Gordillo
R., Liu T., Odle A.K., Childs G.V., Zhang N., Kusminski C.M.,
Elmquist J.K., Williams K.W., An Z., Scherer P.E. Partial leptin
reduction as an insulin sensitization and weight loss strategy. Cell
Metab. 2019;30(4):706-719.e6. DOI 10.1016/j.cmet.2019.08.005

